# Phase Meter Calibration at NBS

**DOI:** 10.6028/jres.093.006

**Published:** 1988-02-01

**Authors:** Raymond S. Turgel

**Affiliations:** National Bureau of Standards, Gaithersburg, MD 20899

**Keywords:** calibration, calibration uncertainty, direct digital synthesis, phase angle, phase angle standard, phase meter

## Abstract

To provide a phase meter calibration service, a phase angle calibration standard has been developed at NBS. This standard is a signal generator with two sinusoidal outputs and uses direct digital synthesis to generate the signals. The phase angle between the two sinusoids is determined by the input parameters in the calculation of the sets of digital values from which the analog output is synthesized. An auto-zero compensation mode corrects for residual phase differences in the two output channels. The phase resolution is better than 0.002° over a frequency range from 2 Hz to 5 kHz and 0.005° from 5 to 50 kHz. Phase meter calibration data are fitted to a linear model from which appropriate corrections for the phase meter readings can be derived. Statistical treatment of the data provides an estimate of the uncertainty of the corrected phase meter readings relative to the phase angle calibration standard.

## Introduction

Phase angle is the quantity that describes the time relationship between waveforms of two ac signals of the same frequency. Phase meters that measure this angle have a wide range of applications in industry and the laboratory. In electric generating stations phase meters are used to check the distribution circuits; in navigational equipment, measuring the phase angle provides a method of determining the bearing with respect to a ground coordinate system; in industry, many positional displacements are monitored by determining the electrical phase angle, and phase shift is an important design parameter of electronic amplifiers and other circuitry. While developments of phase meters have moved along with progress in instrumentation technology, until recently, accurate phase meter calibration had to contend with more traditional and rather time consuming procedures, requiring many steps to make a single measurement.

## Phase Meter Calibration

To establish a precision phase meter calibration service at NBS, it was concluded that refinement of traditional methods did not seem to offer possibilities for the desired significant improvement. To improve the state of the art, a new approach was needed. In an ideal calibration arrangement, the meter to be calibrated is connected directly to a calibrator which can be set for the appropriate test conditions. The meter output is then compared to the value furnished by the calibrator (standard). In many cases this simple set-up cannot be realized in practice, but for the phase meter calibration the aim has been to approach this simple concept as closely as possible.

For the precision calibration of a phase meter, two sinusoidal signals of the same frequency are needed that have very low distortion and are displaced by a very stable, precisely known phase angle. The low distortion sinusoidal test signal is desirable because the phase angle, strictly speaking, is defined only if the two waveforms are identical, and it is extremely difficult to ascertain the exact equality, in both amplitude and relative phase, of the components of complex waveforms. Thus, in practice, precision phase measurements are meaningful only for signals with low distortion. Various designs of phase meters discriminate against harmonics in different ways, so that a distorted signal may yield measurement results that differ from one type of meter to another, a condition which is clearly unacceptable for a general calibration service.

## Principle of the Phase Angle Calibration Standard

With the above consideration in mind, the test signal requirement can best be fulfilled by a special high purity, dual-channel signal source and some highly accurate means to determine the phase angle between the two outputs. Prior experience with digital signal processing circuitry indicated that application of digital techniques to analog measurements would provide advantages that could not easily be achieved by purely analog methods. By combining the inherent stability and predictable precision of digital calculations with digital-to-analog converters, it is possible to generate the desired pair of pure sinusoidal waveforms necessary for the calibration of a phase meter and, at the same time, determine the phase angle with the desired accuracy.

The output of the dual-channel signal generator is provided by a pair of fast digital-to-analog converters that translate numerical input data into analog output voltages. The required numerical values for each waveform are calculated point-by-point, using a high-speed microprocessor, and then converted into corresponding analog output voltages. The resulting discrete output voltages form a stepped waveform which is then passed through smoothing filters and amplified to provide a sinusoidal signal. This method of direct digital synthesis is analogous to graphical construction of functions by plotting them point-by-point.

**Figure 1:**
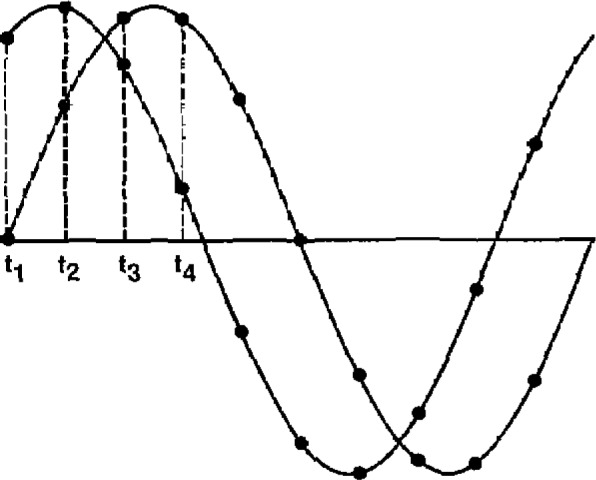
Waveform synthesis with uniform sampling.

The desired phase relationship of the two output signals is achieved by carrying out the digital-to-analog conversion of each point on one waveform simultaneously with the conversion of a corresponding point on the other waveform (see fig. 1). Since each waveform is uniquely determined by the set of calculated numerical values that make up the sample points, the waveforms can be displaced relative to one another to yield the desired phase angle, by choosing appropriate parameters for the calculations. Hence, the phase angle is essentially determined by a mathematical algorithm and is, therefore, accurately known and drift free. When generated by this method, the phase angle is not affected by the limitations and problems associated with analog phase shifters which are dependent on the stability of their electronic components and the operating frequency. Additional advantages of direct digital synthesis are the excellent repeatability, stability, and low distortion of the waveforms.

## Phase Standard Prototype

**Figure 2:**
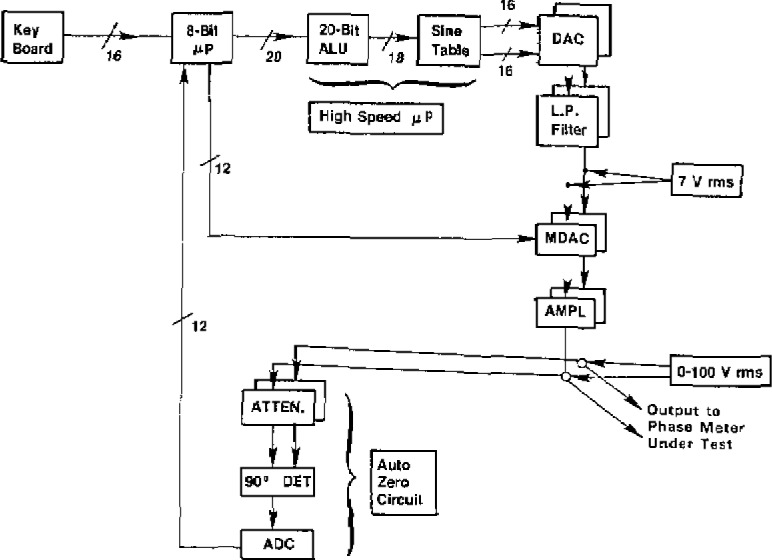
Functional block diagram of NBS phase standard.

Based on the principles of signal generation outlined above, NBS built three prototypes of a phase angle calibration standard, one for its own use, the others for the Department of Defense. The construction and circuits of the NBS Phase Angle Standard are described in detail in the literature [1–4]. The standard uses two embedded microprocessors (see fig. 2), a high-speed, bit-slice microprocessor to generate the waveforms and a second conventional microprocessor to control the rest of the system, performing functions such as setting the desired test parameters (phase angle, frequency, and amplitude in each channel), as well as performing auxiliary functions associated with a parameter display, error correction, and communication with the IEEE-488 instrumentation bus.

### Waveform Generation

All calculations connected with the direct digital synthesis of the two output waveforms is carried out in a bit-slice, 20-bit wide microprocessor. Twenty bits (including a sign bit as most significant hit and a guard bit as least significant bit) are needed to obtain slightly better than 0.002° resolution in phase angle over a ±999.999° span. The location along the time axis, *t_i_*, of each sample point on the waveform can be expressed as an equivalent phase angle relative to the positive-going zero crossing of the sinusoid (see fig. 1). These angles are calculated by successively adding a constant angular increment (equivalent to uniform sampling in time) to some selected starting value. The size of the angular increment is determined by the number of sample points per waveform.

At each point, the instantaneous amplitude is computed using the sine function with the corresponding equivalent angle as argument A special hardware module provides these angle-to-sine conversions, with 16-bit output resolution, in less than 400 nanoseconds, which allows all computations to be carried out in real time for signal frequencies up to 5 kHz. For signal frequencies from 5 to 50 kHz, where the number of samples for each period of the waveform never exceeds 512, sets of numerical data for the two waveforms are calculated only once for each change of frequency or phase angle and are then stored in memory. From memory, the sample data can be transferred rapidly, at rates from two to four million per second, to 12-bit, high-speed analog-to-digital converters.

The number of sample points per waveform is chosen so that the rate at which the numerical data are converted into corresponding voltages remains in a band from 200 to 400 kHz for output frequencies up to 5 kHz and in a band from 2 to 4 MHz above 5 kHz. With these choices a single low-pass filter for the 16-bit (low-frequency) channel and a single low-pass filter for the 12-bit (high-frequency) channel is sufficient for all output frequencies. To provide acceptable waveform purity the minimum number of samples is 64 (at 5 and 50 kHz, respectively). To keep within the constraints of the conversion rate, the number of samples is doubled when the output frequency selected is halved. Thus, at 2 Hz the waveform is reconstructed using 131072 (=64×2^11^) samples. The large number of sample points results in excellent spectral purity, but makes storing the set of data uneconomical and thus requires real-time computation.

### Frequency Selection

The frequency band of the phase standard extends from 2 to 50 kHz, adjustable in steps of 1 Hz for frequencies up to 5 kHz and adjustable in steps of 10 Hz from 5 to 50 kHz. Operationally, the two frequency ranges differ, although the difference is essentially transparent to the user. In the lower range data computed in real-time are applied to 16-bit digital-to-analog converters, and in the upper range, as explained above, stored data are applied to higher speed 12-bit converters. In either case the timing pulse that transfers the data to the converters is obtained from a frequency synthesizer which is independent of the microprocessor clock. This method permits synchronization of the output signal with an external source. The frequency and phase information entered through the keyboard or via the IEEE-488 bus, is transferred to the waveform generating computer at the beginning of every cycle of the output signal, thus providing almost instantaneous response. Reinitializing the waveform generating computation at the beginning of every cycle of the output also prevents spurious errors from being propagated for more than one cycle.

### Amplitude Control

Phase meters are often used with signals of unequal amplitudes at the two inputs. It is therefore desirable to be able to test and calibrate them under those conditions. The phase angle standard uses programmable attenuators ahead of amplifiers which are capable of supplying a seven-volt rms output. For voltages up to 100 volts, an additional internal, fixed-gain amplifier is switched into the circuit. The amplitude of each output channel is independently controllable. For those phase meters requiring current inputs, auxiliary transconductance amplifiers [5] capable of supplying up to five amperes can be connected externally.

### Automatic Phase Compensation (Auto-Zero)

An important feature needed to assure the accuracy of the NBS phase angle calibration standard is an automated procedure that checks the output phase angle and corrects for small residual differential phase offsets. These phase offsets arise because, in practice, the amplifier and filter circuits in the two output channels, although carefully matched, cannot be made to be truly identical. Using circuitry internal to the phase angle standard, the procedure establishes a true 90° phase angle as a reference, compares it to the nominal 90° phase angle of the output signals, and, in several iterations, calculates and applies a phase angle correction to the waveform generating processor.

The method is based on a quadrature phase detector circuit [6] which has a voltage output proportional to the cosine of the phase angle between the two inputs and which is nominally zero when the input signals are exactly 90° out of phase. To eliminate any errors that might be introduced by the phase director itself, during every iteration measurements are made at +90° and −90° and again with the signal channels interchanged. This set of four measurements compensates for dc offsets as well as phase offsets in the detector, effectively making it behave like an ideal component.

The final value of the correction is stored in the memory of the microprocessor and is then automatically applied to any selected phase angle settings used for the calibration of the phase meter. The auto-zero compensation needs to be redetermined every time the amplitude of either channel is changed, or if the frequency is shifted between ranges (see below). Within each frequency range, the compensation is adjusted by interpolation.

### Accuracy

In an ideal phase angle standard there is a one-to-one correspondence between the phase angle of the output signals and the setting of the standard. In the actual implementation of the standard, there will be departures from this ideal relationship. These departures can be attributed to several causes, some of which are predictable, while others are subject to the random effects of noise.

One of the predictable effects is the rounding error. The keyboard entry of the phase angle is in decimal form, in steps of 0.001°, while the internal calculations are carried out in a binary number system with an equivalent resolution of 0.00137…°. Therefore, not every keyboard setting corresponds to a unique binary value for the angle. Furthermore, at the upper limit of the frequency range, when using 12-bit converters and 64 sample points per waveform, it can be shown [4] that the phase angle resolution of the output signals, which is theoretically possible under these conditions, is closer to 0.005°.

Another contributing factor to the departure from the ideal model of the phase standard is the difference between the actual and theoretical response characteristics of the particular digital-to-analog converters used. Non-linearities in these characteristics result in slight waveform distortions which degrade the accuracy of the phase angle. Although, by measuring the converter response carefully, it might be possible to evaluate the error at any particular phase angle, the number of calculations would be too large to make this practical as a general approach. Instead, an estimate of the limits of the departure from linearity of the relationship between output phase angle and setting of the standard can be obtained by a limited series of measurements. From these an estimate of the uncertainty in the assignment of the phase angle of the output of the standard can be derived.

One of the random factors that influence the phase standard accuracy is a residual phase offset that remains after the auto-zero procedure attempts to compensate for offsets due to small mismatches in the amplifiers and filter characteristics. The determination of the value of this auto-zero correction by the system is dependent on feedback to the signal generating processor of numerical data from the results of repeated measurements with the quadrature detector. Since each measurement is affected by noise in the system, the auto-zero compensation may not settle to the same endpoint every time, and it therefore introduces some random uncertainty into the value of the phase angle. The magnitude of that uncertainty depends on the amplitudes of the signal as well as on the frequency.

At frequencies below 1 kHz the corrections are relatively small, only slightly larger than the angular resolution of the standard. With increased frequency, all of the effects are more pronounced. A fuller discussion is given in [2] and [4]. Specifications shown in table 1 are based on experimentally determined accuracies of the three prototype phase standards built at NBS.

### Accuracy Verification

An important part of any reference standard is the verification of its own accuracy. To understand what is involved in the case of a phase angle standard, one has to consider the “three-dimensional measurement space" that encompasses phase angles, frequencies, and amplitudes. There is an infinity of possible test points that obviously cannot all be covered. The two main parameters that need checking on various ranges are the linearity and the offset compensation.

Of these, the offset compensation is most readily measured. A bridge circuit can be constructed in which the two signal generating channels of the standard, which have a common ground terminal, are configured as two arms of the bridge and two suitable impedances, connected in series across from one output terminal of the standard to the other, comprise the other two arms. A null detector is then connected from the junction of the two impedances to the ground terminal of the standard. Since two of the arms are active (and coherent) signal sources, no other supply is needed. To improve the signal-to-noise ratio the detector is tuned to the operating frequency. For the lower frequency range, the impedances consist of the windings of an inductive divider, and for the higher frequency range two three-terminal, variable air capacitors are used.

To balance the bridge both the ratio of the external impedances and the relative phase of the two output signals from the phase angle standard must be adjusted. The angular offset from the nominal 180° is the phase error of the standard. Additionally, the quadrature phase error of the standard can be checked with an external quadrature detector using a method similar to that of the auto-zero procedure.

Linearity tests can be carried out by a variety of procedures. In the vicinity of 90° and near the 180° point, using the quadrature detector or the bridge circuit, respectively, off-null voltage readings on the detector should be proportional to the angular deviation from 90° or 180°. This holds true as long as the angular deviation is small enough so that the sine of the (deviation) angle is equal to the angle (in radians), within the required accuracy. A more general but very time consuming method, applicable at any phase angle, makes use of a fixed (≃ 1°) and a variable phase (0° to 360°) shifter inserted into one arm of a 180° bridge. At various, phase angles, the bridge is first balanced with only the variable phase shifter in the circuit. Then, with both the fixed and the variable phase shifters in the circuit, the incremental phase adjustment of the standard needed to rebalance the bridge is determined. A somewhat simpler version of this method makes use of the independent phase adjustment of the two output channels, instead of added phase shifters, to obtain bridge balances with one channel set at a phase angle *ϕ* (relative to an internal reference common to both channels) and the other channel at (*ϕ* + 180° + δ), where δ represents the linearity error. Detailed discussions can be found in references [3] and [4].

## Phase Meter Calibration

Using the NBS phase angle standard, phase meter calibrations can be carried out with significant savings in time and effort even in the manual mode. To make full use of its potential, however, the calibration system should be operated from an instrument controller (computer) via the instrumentation (IEEE-488) bus.

For its phase meter calibration service, NBS has developed a phase meter calibration program that combines automatiac control of the phase standard with statistical analysis of the calibrations results. The projected service will be described in an NBS Special Publication which is in preparation [7].

The phase meter is directly connected to the phase standard if the meter ranges match the output capability of the standard (0.5 to 100 volts rms, 2 to 50 kHz). If higher voltages (240 volts rms) or currents (0.05 to 5 amperes) are required, special auxiliary amplifiers with known phase offsets are added. On every selected range, phase angle test points are selected by the operator. Experience has shown that satisfactory accuracy can be obtained with 12 test points covering the span from. 0° to 360°.

The program then proceeds with the calibration measurements by setting the phase angle standard to the desired value. To minimize the effect of time dependent drifts, the order of the test points (including repeat measurements) is randomized, and when the sequence is completed, every selected phase angle will have been measured at least four times. The phase meter reading can be entered manually through the controller keyboard, or if provided for, automatically via the IEEE-488 bus. All data are stored on magnetic disk or tape.

Parameters derived from the replicate calibration readings make possible a statistical test of whether the phase meter characteristic can be represented by a linear relationship, or is significantly non-linear. Further statistical tests establish whether the modeled linear response characteristic significantly differs from the ideal. On the basis of this statistical evaluation, suitable calibration corrections can then be assigned to bring the meter readings into agreement with the standard [8].

The modeled response characteristic (calibration curve) derived from the statistical treatment of the calibration data, generally provides a better basis for computing this corrections than that obtained by simply averaging data from each test point. The NBS Calibration Report provides full information on the computation for the corrected values, as well as some statistical background.

## Other Applications of the Phase Angle Standard

Because of its excellent phase and waveform stability, the phase standard has found applications not immediately related to its phase meter calibration function, and it has formed the basis for further specialized instrumentation development. For instance, with auxiliary voltage and current amplifiers, the phase standard can be used as a stable, easily adjustable signal source for checking wattmeters and watthour meters [9,10]. It can also be used to test various signal analyzers that need a phase-variable reference. In another application, the special properties of the two-signal, phase-variable source provided by the standard have resulted in the development of new techniques and new configurations for ac impedance bridge measurements.

## Trends in Precision Measurement Instrumentation

In recent years, developments in precision instrumentation have shifted the burden of making successful measurements more and more from man to machine. Instead of relying entirely on personal skills and conscientiousness of the experimenter, the execution of the task has been transferred to the software of a system using external instrument controllers and embedded microprocessors. In line with this trend, the responsibility for the ultimate accuracy has shifted, at least in part, from the person performing the measurement to the instrument designer and software engineer who devise the measurement process. The aim in this development has been to eliminate the tedium of repetitive manual operations and prevent associated fatigue induced errors. At the same time, the overall efficiency and accuracy can be improved by refinements in the measurement process.

However, this increased reliance on the instrumentation is sound only if the instruments perform flawlessly, since the skilled judgement of the experimenter ostensibly has been replaced by the automated process. To assure continued success in meeting the objective of accuracy in measurements when using automated systems, greater importance must be given to the activities which support the instrumentation. Calibration is one of these support activities, assuring that the results obtained conform to accepted measurement standards and that the instrument is functioning properly.

Traditionally, calibration has been a very labor-intensive activity. In many areas, particularly those where the volume of calibrations is high, older manual laboratory methods have already been successfully replaced by automated electronics; for instance, the replacement of volt boxes and potentiometers by electronic voltage standards for voltage calibration. In other areas, where the economic pressure is perhaps less pronounced, innovations in calibration of phase meters is one of those areas.

## Figures and Tables

**Table 1:** Performance specifications based on measurements of NBS prototypes

Phase Angle:	
Range	−999.999 to +999.999 degrees
Resolution	0.0014 degrees (1 part in 2^18^)
Systematic uncertainty (equal amplitude)	
at 60 Hz	0.003 degrees
at 400 Hz	0.004 degrees
at 5 kHz	0.008 degrees
at 15 kHz	0.016 degrees
at 30 kHz	0.027 degrees
at 50 kHz	0.040 degrees
Systematic uncertainty (amplitude ratio 7:1)	
at 60 Hz	0.004 degrees
at 400 Hz	0.006 degrees
at 5 kHz	0.011 degrees
at 50 kHz	0.080 degrees
Output frequency:	
Range	2 Hz to 50 kHz
Resolution	1 Hz up to 5 kHz; 10 Hz above 5 kHz
Accuracy	0.06%
Stability	1 ppm
Output amplitude:	
Effective range	0.5 to 100.0 V rms
Resolution	steps of approx. 2 mV, up to 7 V steps of approx. 24 mV, 7 to 100 V
Accuracy	0.1%
dc offset voltage	< 0.5 mV for outputs to 7 V rms < 5 mV for outputs 7 to 100 V rms
